# Differences between mates at the *TLR1Lb* locus are associated with lower reproductive success in a long-lived seabird

**DOI:** 10.1038/s41598-024-77750-7

**Published:** 2024-12-30

**Authors:** Marianne Gousy-Leblanc, Thomas Merkling, Lila Colston-Nepali, Emma Lachance Linklater, Kyle H. Elliott, Vicki L. Friesen

**Affiliations:** 1 Department of Natural Resource Sciences, McGill University, Ste-Anne-de-Bellevue, QC Canada; 2 Department of Biology, Queen’s University, Kingston, ON Canada

**Keywords:** Brünnich’s guillemot, Immunity, Single-nucleotide polymorphism, Thick-billed murre, Toll-like receptors, Ecological genetics, Molecular ecology, Animal behaviour

## Abstract

Genetic diversity can influence fitness components such as survival and reproductive success. Yet the association between genetic diversity and fitness based on neutral loci is sometime very weak and inconsistent, with relationships varying among taxa due to confounding effects of population demography and life history. Fitness-diversity relationships are likely to be stronger and more consistent for genes known to influence phenotypic traits, such as immunity-related genes, and may also depend on the genetic differences between breeding partners. We recorded breeding success of individuals and breeding pairs over 20 years to evaluate the relationships between reproductive success and both neutral genetic variation (using 7,830 single nucleotide polymorphisms) and functional variation (four toll-like receptor [TLRs] loci) with reproductive success in thick-billed murres (*Uria lomvia*). Individual genetic diversity (both neutral and functional) was unrelated to reproductive success, but surprisingly, successful multi-year reproductive success decreased with the genetic difference between breeding partners at *TLR1Lb*. This result may be due to an advantage of specific alleles at *TLR1Lb*. This study is one of few addressing both individual genetic variation and genetic similarity between mates at both neutral and functional variation in a long-lived bird.

**Supplementary Information:**

The online version contains supplementary material available at 10.1038/s41598-024-77750-7.

## Introduction

Understanding the genetic basis of fitness variation within populations is a central objective for evolutionary biologists^1,2^. Of particular importance is individual genetic variability (genetic diversity) as it allows species to adapt to changes (e.g. environmental) and limit inbreeding depression^3^. Genetic diversity can be directly related to variation in fitness components such as survival and reproductive success^4–8^. For example, maternal heterozygosity (a measure of genetic variability) was positively associated with offspring body size in great tits (*Parus major*) presumably because highly heterozygous mothers can allocate more resources to their progeny during development^9,10^. As another example, on Mandarte Island, male song sparrows (*Melospiza melodia*) that have low heterozygosity (due to higher inbreeding) had lower immune responses and song repertoires, leading to fewer mating opportunities and lower reproductive success than males with higher heterozygosity^11^. However, the relationship between fitness and genetic diversity based on neutral loci is sometimes very weak and inconsistent, with relationships varying among taxa due to the confounding effects of demography and life history^1,12–14^. The relationship between fitness and genetic diversity at genes known to influence phenotypic traits, such as immunity, may be stronger but is less frequently studied (Table 1).

**Table 1:** Summary of published studies on the association between immune gene diversity and fitness in birds.

Species	Gene	Fitness proxy	Association	References
Attwater’s prairie-chicken (*Tympanuchus cupido attwateri*)	MHC + TLR	Survival	+ specific allele	^5^
Barn owl (*Tyto alba*)	MHC	Reproductive success	None	^20^
Black-headed gull (*Chroicocephalus ridibundus*)	TLR	Phenotypic trait	+	^60^
Collared flycathchers (*Ficedula albicollis*)	MHC	Lifetime reproductive success	None	^47^
Common yellowthroat (*Geothlypis trichas*)	MHC	Survival	+	^83^
Dunnock (*Prunella modularis*)	TLR	Number of chicks produced	+ TLR3	^84^
Egyptian vulture (*Neophron persnopterus)*	MHC	Reproductive success	+	^4^
Eurasian coot (*Fulica atra*)	MHC	Reproductive performance & phenotypic quality	-	^31^
Great reed warbler (*Acroceplalus arundinaceus*)	MHC	Chick recruitment	+/- depending on sex	^85^
Leach’s storm-petrel (*Oceanodroma leucorhoa)*	MHC	Reproductive success	+	^50^
Pale-headed Brushfinch (*Atlapetes pallidiceps*)	TLR	Survival	-	^86^
Song sparrow (*Melospiza melodia*)	MHC	Survival	-	^87^
Stewart Island robin (*Petroica australis rakiura*)	TLR	Survival	+ TLR4	^28^
Thick-billed murre	TLR	Reproductive success	+ *TLR1Lb* similar for mates	This study

Immune-related genes, such as genes for toll-like receptors (TLRs) and the major histocompatibility complex (MHC), are regarded as essential for individual fitness^15^. The relationship between MHC heterozygosity and diverse fitness proxies has been studied in a wide variety of species (e.g. migratory caribou *Rangifer tarandus*^16^; three-spined sticklebacks *Gasterosteus aculeatus*^17^; Alpine chamois *Rupicapra rupicapra*^18^; house mice *Mus musculus musculus*^19^; barn owl *Tyto alba*^20^). Compared to MHC, TLRs genes are understudied, and the link between genetic diversity at TLRs and fitness has been investigated only relatively recently (Table 1). TLRs are central components of the vertebrate innate and acquired immune systems (immune response through intracellular signaling^21,22^). They play a crucial role in frontline defense against a wide diversity of pathogens and diseases^23,24^. TLRs are classed as pattern recognition receptors (PRRs) that form a direct molecular interface between the host and a particular pathogen-associated molecular pattern (PAMP). The host is able to detect infection and trigger an immune response against a variety of microbial ligands from viruses, bacteria, parasites and fungi^23,25,26^.

TLR genes show moderate to high levels of diversity in wild populations and this variation has been associated with resilience to infections^27–29^. Thus, possessing two different alleles at the same TLR locus should allow hosts to sense a broader spectrum of PAMP structures, thereby triggering an immune response against a greater range of foreign pathogens than in homozygous individuals^30^. Higher heterozygosity across multiple TLR loci, or even within a single TLR locus, should be related to higher fitness due to more efficient protection against multiple pathogens^25^ (Table 1).

The strength of the association between diversity in immune genes and fitness varies among studies, with studies reporting negative^31^, positive^4^ or intermediate^32^ associations all being reported (Table 1). These discrepancies may arise either because the fitness costs and benefits of immune gene diversity differ among individuals depending on their exposure and immune responses to pathogens^33^, or life histories differ among individuals or species^1^. A low genetic diversity at TLR genes could indicate that an individual is more likely to be threatened by changes in the environment that may expose it to new or more diseases^34^. Conversely, an overly strong immune system (high genetic diversity) could be energetically costly and affect body mass or survival^16^, or generate autoimmune diseases^35^. Consequently, an intermediate level of diversity could be ideal to optimise fitness in some cases^17^.

Relationships between individual heterozygosity and either reproductive success or fitness components may also be inconsistent because reproductive success depends on the genetic compatibility (e.g. genetic similarity) between mates as well as the genotype of each individual (e.g. blue tits *Cyanistes caeruleus*^36^; great tits *Parus major*^37^). This may be especially important in monogamous species with long-term pair-bonds, as the choice of mate may strongly affect reproductive success throughout an individual’s lifetime^38^. Positive relationships between parental genetic similarity and reproductive success have been found in systems where mating with a genetically similar mate can either prevent disruption of co-adapted genes or confer a benefit in terms of kin selection^39^. Conversely, some studies found significant negative relationships between parental similarity and reproductive success because of the fitness cost of producing homozygous offspring^38,40^.

**Fig. 1:**
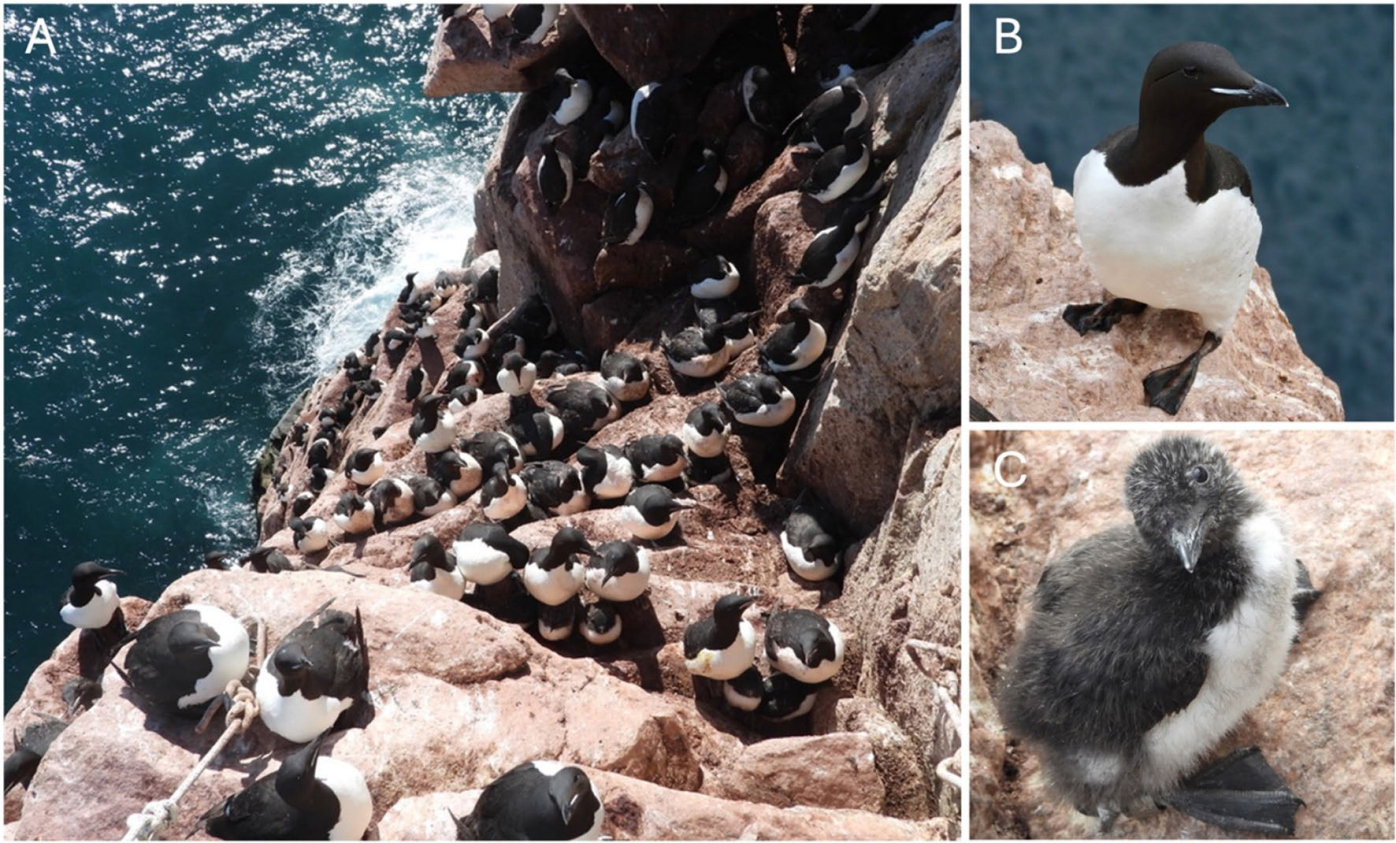
(**A**) Cliff-nesting thick-billed murres from Coats Island (Nunavut, CA) during the chick-rearing. (**B**) An adult thick-billed murre and (**C**) A thick-billed murre chick.

We used long-term data on breeding success of individuals and breeding pairs to evaluate the relationship between genetic variation and fitness in a long-lived seabird. The thick-billed murre (*Uria lomvia*) is a colonial cliff-nesting seabird with obligate biparental care and social and genetic monogamy (Fig. 1). They raise only one chick per breeding attempt. Mosquito parasitism can cause up to 30% of pairs to fail to fledge a chick in some years^41,42^, and the high rate of blood parasitism means that the robustness of the immune systems of both chicks and adults is likely important for reproductive success. Murres exhibit high fidelity to their breeding partner^43^ and low extra-pair paternity (7%)^44^. They are also philopatric to their nesting sites, which facilitates long-term monitoring of individuals and breeding pairs.

Here, we evaluated the relationships between both neutral genetic variation (using 7,830 single nucleotide polymorphisms [SNPs]) and four TLR loci with reproductive success over 20 years of monitoring at an Arctic colony. We hypothesized that individual reproductive success will be highest at an intermediate level of genetic diversity as a too high level of genetic diversity could be costly and a low level of genetic diversity could be disadvantageous. At the breeding pair level, we expected that reproductive success should be positively correlated with genetic difference between partners as a high genetic difference between partners could favor the genetic diversity of the offspring.

## Results

### Analyses of individual variation

The final dataset for the neutral (RADseq) variation for individuals included 7,830 SNPs for 92 individuals. We had data for 608 breeding attempts for annual reproductive success analyses and 92 for multi-year (Table 2A). We did not find a correlation between either MLH or F and either annual or multi-year reproductive success. In all cases, the most likely model was the null model (Supplementary Table 1). The final dataset for the functional variation included four TLR genes (Table 2B), 635 breeding attempts and 127 individuals (Table 2A). We did not find a correlation between multi-locus heterozygosity (MLH), homozygosity by locus (HL), proportion of heterozygous loci (PHt), internal relatedness (IR) or heterozygosity at TLR genes and either annual or multi-year reproductive success (Supplementary Tables 2, 3). Neither the mean number of amino acid substitutions nor the total number of amino acid substitutions over the four loci were associated with either annual or multi-year reproductive success (Supplementary Table 4).

**Table 2:** (A) Sample size for individual and breeding pairs analyses for both type of genetic variation. RS = reproductive success (B) polymorphisms at four TLR genes in thick-billed murres at Coats Island.

(A)					
Sample size	Neutral variation	Functional variation			
Individuals	92	127			
Annual RS	608	635			
Multi-year RS	92	127			
Breeding pairs	44	62			
Annual RS	169	205			
Multi-year RS	44	62			

(B)					
Locus	Fragment size (bp)	Number of amino acids	Number of amino acid variants	Number of alleles	Number of genotypes
*TLR1La*	129	43	2	3	4
*TLR1Lb*	972	324	20	27	51
*TLR4*	852	284	8	13	22
*TLR5*	1278	426	15	18	31

### Analyses of breeding pairs

The final dataset for the neutral (RADseq) variation for partners included 169 breeding attempts over 44 breeding pairs (Table 2A). We did not find a correlation between genetic similarity and either annual or multi-year reproductive success (Supplementary Table 5).

**Fig. 2:**
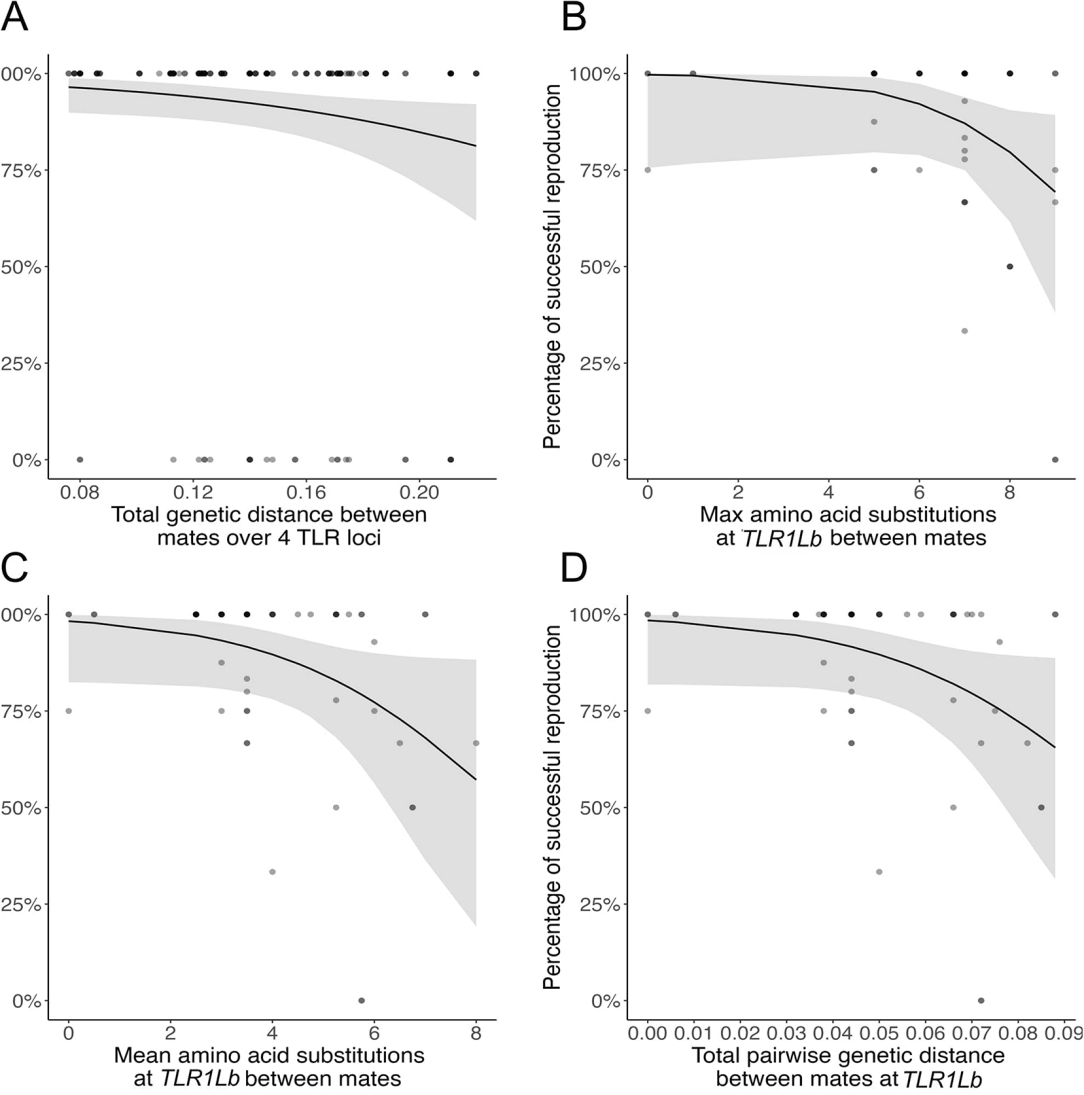
(**A**) Probability of successful reproduction as a function of the total pairwise genetic distance between mates over four TLR loci. Percentage of breeding attempts (multi-year reproductive success) that were successful as (**B**) a function of maximum and (**C**) mean number of amino acid differences at *TLR1Lb* between individuals in a breeding pair (**D**) as a function of the total pairwise genetic distance at *TLR1Lb* between individual thick-billed murres in a breeding pair.

The final dataset on differences in functional genetic variation between partners comprised 205 breeding attempts over 62 breeding pairs (Table 2A). Maximum and mean amino acid substitutions between breeding partners at each of the TLR loci did not correlate with annual reproductive success (Supplementary Table 6a). The most likely model for the annual reproductive success included the total pairwise genetic difference between mates over the four TLR loci (Supplementary Table 7a). The probability of having a successful breeding attempt decreased with an increase in genetic distance between mates over all four TLR loci (estimate = -12.80; 95% CI = -24.67, -0.93; R^2^c = 0.056; R^2^m = 0.153; Fig. 2A).

For the multi-year reproductive success for breeding partners, the most likely models included maximum and mean amino acid substitutions at *TLR1Lb* (Supplementary Table 6b). The proportion of reproductive attempts that were successful decreased with an increase in maximum amino acid substitutions (estimate = -0.558; 95% CI = -1.250, -0.018; R^2^ = 0.163; Fig. 2B) and mean amino acid substitutions (estimate = -0.467; 95% CI = -0.998, -0.006; R^2^ = 0.155; Fig. 2C) between mates at *TLR1Lb*. The proportion of reproductive attempts that were successful also decreased with the pairwise genetic distance at *TLR1Lb* (estimate =-39.81; 95%CI = -86.03, -0.34; R^2^ = 0.153; Fig. 2D).

## Discussion

We evaluated the association of both neutral and functional genetic diversity with reproductive success in a long-lived seabird whose reproductive success is strongly impacted by blood parasitism^41,42^. The present study is one of the few addressing both individual genetic variation and genetic similarity between mates at both neutral and functional loci. Surprisingly, multi-year reproductive success decreased with greater genetic difference between partners at *TLR1Lb*  in the thick-billed murre. We did not find an effect of individual heterozygosity on reproductive success with either type of genetic diversity.

### Analyses of individual variation

Contrary to our hypotheses, we did not find a correlation between individual genetic variation and either annual or multi-year reproductive success. For neutral variation, the absence of a relationship is not surprising. Correlations between heterozygosity at neutral loci and fitness proxies often entail small, declining and/or inbred populations (e.g. ^4,8,13,14^). In contrast, the murre colony at Coats Island is large (~ 30,000 pairs), and stable or slightly increasing^45^. According to a quantitative review of heterozygosity-fitness correlations in animal populations, less than 1% of the variance in fitness traits is explained by heterozygosity metrics^1^.  Some other studies also failed to find associations between genome-wide heterozygosity and reproductive success (e.g. Eurasian coot *Fulica atra*^31^; collared flycatchers *Ficedula albicollis*^46^). With 7,830 SNPs, we had more precise estimates of the genome-wide heterozygosity and more power to detect an effect than is achievable using a small set of microsatellites or a pedigree-based inbreeding coefficient^14,47–49^.

In terms of functional variation, the absence of a significant association between individual diversity at TLR loci and reproductive success in thick-billed murres is comparable to a study of the collared flycatcher, where no association between individual MHC diversity and lifetime reproductive success was found^46^. However, we could only use fledging success at 14 days as our reproductive success measure, rather than recruitment to adulthood, because recruitment is difficult to measure in murres. Other reproductive success metrics, such as hatching success or recruitment, could have provided different results, as in previous studies on seabirds. For example, in Leach’s storm-petrels (*Hydrobates leucorhous*), females that were homozygous at the MHC class IIB locus had a lower probability of hatching a chick^50^ and in the common tern (*Sterna hirundo*), multilocus heterozygosity at 15 microsatellite markers was not correlated with fledging probability, but was positively correlated with recruitment probability^32^.

We also did not detect an effect of sex on the relationship between heterozygosity and reproductive success. Although both parents invest in rearing the chick to fledging, the male stays with the chick after fledging. The correlation between heterozygosity and fitness proxies is often different between males and females. For example, in European shags (*Phalacrocorax aristotelis*) a positive correlation between heterozygosity, reproductive performance and survival was only detected in females^8^; in Leach’s storm-petrels, only the heterozygosity of the female correlated with chick mass^14^; and in the common tern, annual fledgling production was negatively correlated with heterozygosity in males and highest in females with intermediate heterozygosity^32^.

### Analyses of breeding pairs

Numerous studies have investigated the effect of relatedness between partners on reproductive success (e.g. ^13,51,52^), but only a handful tested the effect of genetic difference in immune genes between breeding partners. For example, breeding success was higher for breeding pairs with higher difference in their MHC alleles in the Egyptian vulture (*Neophron percnopterus*^4^). Our finding of higher reproductive success for mates that are similar at *TLR1Lb* was the opposite relationship of our expectation. Other studies of monogamous long-lived seabirds have found a negative relationship between similarity of mates at neutral loci and reproductive success^38,40^. However, some studies of birds have found that individuals choose to mate with genetically similar partners (e.g. great frigatebirds *Fregata minor*^53^; house sparrow *Passer domesticus*^39^ and rock pigeon *Colimba livia*^54^. Possibilities explaining these results are that the choice of a genetically similar mate is maintained by a benefit to low-level inbreeding; mating with a related partner is advantageous as it increases the parent’s inclusive fitness; or the estimated level of inbreeding is based on selectively neutral variation^53,54^. The Eurasian coot, showed a negative association of functional MHC diversity with phenotypic quality and reproductive performance^31^. However, in our study we only found an association between reproductive success and genetic similarity at one gene, *TLR1Lb*, suggesting that homozygosity at *TLR1Lb* is adaptive.

The four TLR loci that we targeted detect different pathogens: *TLR4* detects host self (e.g. fibrinogen or endogenous phospholipids) or nonself (e.g. lipopolysaccharide) ligands; *TLR5* detects bacterial flagellin, gram negative bacteria and hematozoans; and *TLR1* binds di- and triacylated lipoproteins anchored in the cell wall of bacteria, fungi, and parasites ^22,25,27,55^. The extracellular receptor of *TLR1Lb* is known to initiate immune responses against many common avian pathogens such as mycoplasmas, *Pseudomonas aeruginosa*, *Salmonella* spp. and *Chlamydia* spp^56–59^. Specific alleles at *TLR1Lb* have been associated with variation in fitness in birds. For example, the physiological condition of individual black-headed gulls (*Chroicocephalus ridibundus*) was associated with a specific allele at *TLR1Lb*^60^, and for the Attwater’s prairie-chicken (*Tympanuchus cupido attwateri)* different alleles at *TLR1Lb* correlated with individual survival rates^5^.

While the correlation between breeding success and similarity of mates at *TLR1Lb* was unexpected, the possession of similar alleles at *TLR1Lb* by breeding partners may be advantageous in murres if specific alleles influence reproductive success either positively or negatively. One (or more) allele(s) could affect reproductive success by initiating an immune response to a specific, common pathogen. TLRs have been found to respond to fine-scale spatial variation in pathogen pressure^61^. Alternatively, directional selection on one (or more) alleles at *TLR1Lb* could favor pairings that produce homozygous offspring for the advantageous allele. Positive selection on *TLR1Lb* was found in several penguins species ^62^. However, given the large number of alleles (27 alleles) and genotypes (51), we could not test the hypothesis that one or a few alleles were advantageous or disadvantageous.

## Conclusions

This study is one of few investigating the relationship between both neutral and functional genetic diversity and reproductive success at both the individual and breeding pair level, using long-term data for a bird. Our result of successful multi-year reproductive success decreasing with the genetic difference between breeding partners at a specific TLR locus is quite novel. Studying TLR gene diversity and its relationship to reproductive success helps managers evaluate how murres can cope with changes in their environment. For example, the highly pathogenic avian influenza (H5N1) is a novel threat that emerged recently and strongly impacted murre populations across North Atlantic in 2023. Innate immunity may be more important than adaptive immunity in stemming outbreaks of novel pathogens because pathogen-specific alleles may be absent or occur at low frequencies in the population^5^. Thus, our discovery that a particular TLR gene may be important for reproductive success helps us understand not only the vulnerability of different populations to emerging diseases but also some aspects of mate choice in a long-lived, monogamous organism.

## Methods

### Sample collection

We monitored thick-billed murres on the Coats Island colony in Hudson Bay, Nunavut (Canada; 62°570 N, 82°000 W) between 1991 and 2011 (Fig. 1). This is the longest continuing yearly monitoring available for this colony. Demography and breeding success have been studied at this colony annually since 1984, although with some years of shorter study seasons or no data collection^63^. We measured breeding success by observing each plot daily from mid-June to mid-August. We monitored plots by watching murres from a blind and recorded which individuals had an egg or chick each day^64^. We considered a chick that disappeared after 14 days as a successful breeding attempt^64^. We recorded yearly reproductive success for individuals and breeding pairs, as well as multi-year reproductive success - the proportion of successful reproductive attempts per year that reproduction was attempted. We determined the sex of each murre via copulation position, genetics, or association with a partner of known sex^65^. We collected blood samples from the brachial vein from breeding thick-billed murres. All work with live birds was performed in accordance with ARRIVE guidelines and relevant named guidelines and regulations from an Animal Use Protocol approved by the McGill University Animal Care Committee. Total handling time was under 5 min. All individuals sampled returned to their nest site and they behaved normally after handling. Blood samples were stored in ethanol in a freezer until they were archived at -80 °C at Queen’s University, Canada. We extracted DNA following a standard protease K/phenol-chloroform protocol with ethanol precipitation^66,67^.

### ddRADseq library preparation, sequencing, and processing

We conducted double-digest restriction site-associated DNA sequencing (ddRADseq) as described in Colston-Nepali et al. (2020)^68^. Briefly, we used approximately 500 ng DNA that was double digested using the enzymes MluCl and SbfI (5 units each) for three hours at 37 °C. After digestion, DNA was cleaned using solid phase reversible immobilization (SPRI) beads (Beckman Coulter, Indianapolis, USA)^69^. Adapters were ligated to the DNA fragments, incorporating 48 barcodes and four indexes. Ligated samples were pooled and cleaned again with SPRI beads. The library was then size selected for fragments between 125 and 400 bp (excluding barcodes) with a BluePippin system (Sage Science, Massachusetts, USA). Paired-end sequencing (2 lanes of 125 bp) was completed on an Illumina HiSeq 2500 sequencer at the Centre for Applied Genomics in Toronto.

Loci were also assembled as described in Colston-Nepali et al. (2020)^68^ but using the thick-billed murre reference genome^70^. Only the first SNP per locus was selected. We removed the alleles with a frequency less than 5%. Site depth was filtered to a minimum depth of 10, and a maximum depth of two standard deviations from the mean. We filtered sites to a maximum of 20% missing data, and individuals to a maximum of 30% missing data. Finally, sites were thinned to reduce linkage disequilibrium.

### TLR genotyping

We targeted four TLR genes: *TLR1La*, *TLR1Lb*, *TLR4* and *TLR5*, focusing on the extracellular leucine rich repeat (LRR) region of each gene. We used published primers^27,28^ and primers developed for thick-billed murres^71^ (Supplementary Material Table 8) that were design to amplify the significant functional section of the LRR region of those four genes^27,72^ (see Fig. 4 from^27^ for localization of the targeted sequences). We amplified gene fragments using PCR in 50 µL reaction volumes containing 1x concentration of Multiplex PCR Master Mix^®^ (Qiagen, Venlo, Netherlands), 0.2 µmol each of forward and reverse primers, and 0.01–20 ng DNA. Temperature cycling involved an initial denaturation of 15 min at 95 °C and 40 cycles of 40 s at 94 °C, 75 s at 56 °C, 90 s at 72 °C and a final elongation phase of 10 min at 72 °C. We sequenced PCR products in both directions using Sanger sequencing at the Génome Québec Innovation Center (McGill University, Montreal). We verified whether we amplified the correct target genes by performing Blast searches. The sequences were confirmed and aligned using a Clustal-W multiple alignment algorithm in Geneious Prime 2023.1.1. (Biomatters, Auckland, New Zealand). We trimmed low quality bases at either end so that final sequence lengths for each gene were the same across individuals. We identified heterozygous sites by overlapping peaks of similar height in the chromatograms and assigned International Union of Pure and Applied Chemistry (IUPAC) nucleotide ambiguity codes. For sequences with multiple heterozygous sites, we performed haplotype assignments in DnaSP v5.10.01^73^ using the PHASE algorithm^74^ for 100 iterations after burn-in of 100. We retained only haplotypes that had all heterozygous sites successfully resolved (> 95% confidence level).

We translated phased haplotypes by aligning the sequences with other avian TLR nucleotide sequences available in NCBI non-redundant nucleotide (nr/nt) database. We extracted the functional variation for each TLR by selecting only the sites with non-synonymous mutations using Geneious Prime.

### Analyses of individual variation

#### Neutral genetic variation

For each individual, we calculated multi-locus heterozygosity (MLH) for each SNPs with the *InbreedR* package^75^ in R *v* 4.2.2.^76^, and their inbreeding coefficient (F) using –*het* function in *vcftool*^77^. To test if multi-locus heterozygosity or inbreeding coefficient is correlated with the reproductive success of individuals over the annual breeding attempt, we used generalized linear mixed models (GLMM) with a binomial error distribution and logit link function with the *glmmTMB* package^78^ in R. We performed model selection including an individual’s sex and age, MLH, F, and an interaction between sex and MLH as explanatory variables Some models of the candidate set included a quadratic effect of MLH.  We included year and bird identity as random variables. Fledging success was used as a proxy for reproductive success (i.e., 0 for no fledging, 1 for successful fledging). To test for an association between multi-locus heterozygosity or inbreeding coefficient and multi-year reproductive success of individuals (i.e. the proportion of successful reproductive attempts per year that reproduction was attempted) we used generalized linear models (GLM) with a binomial error distribution and logit link function. We used sex, age, MLH and F as explanatory variables and an interaction between sex and MLH. We used a model selection procedure using the Akaike information criterion (AIC) to determine which models best explained variation in reproductive success. We only kept models with ΔAIC < 2 relative to the best model^79^. If more than one model was selected, we chose the more parsimonious one. The 95% confidence interval was calculated for the most likely model.

#### Functional genetic variation

For each individual for each of the four TLR loci, we computed heterozygosity (0: same alleles, 1: two different alleles). Using the *genhet*^80^ package in R, we calculated the proportion of heterozygous loci (PHt), homozygosity by locus (HL) and internal relatedness (IR) across the four loci. MLH was also calculated across the four loci using *InbreedR*. To test if genetic variation at these TLR genes correlated with annual reproductive success, we used model selection based on GLMM (binomial error distribution and logit link function), with year and bird identity as random variables. MLH, HL, IR and PHt were highly correlated (*r* > 0.98), and so were therefore not included in the same models. The mean number of amino acid substitutions, the total number of amino acid substitutions over the four loci, heterozygosity per locus, sex, age, an interaction between sex and the heterozygosity variables and the quadratic effect of these variables were also included in the candidate set models. We also performed model selection with multi-year reproductive success as the response variable using GLM with the same explanatory variables as above. We selected the best model based on AICc as previously.

### Analyses of breeding pairs

#### Neutral genetic variation

Genetic relatedness between breeding pairs was calculated in *vcftools* (-- relatedness2) using the method from Manichaikul et al.^81^. We standardized (centered and scaled) and used the absolute value to facilitate convergence of models. To evaluate the association between genetic distance between individuals within breeding pairs and annual reproductive success, we performed GLMM (binomial error distribution and logit link). Explanatory variables included genetic relatedness and quadratic effects. The random variables were year and mate identities. To test the influence of the genetic distance between breeding pairs for multi-year breeding success, we performed GLM (binomial error distribution and logit link), but with the proportion of reproductive attempts that were successful as the response variable. Model selection was performed by AICc for both sets of models.

#### Functional genetic variation

We computed the number of amino acid substitutions between alleles for the two individuals in each breeding pair for all four loci. We calculated two amino acid metrics (mean and maximum) from the number of amino acid substitutions between the partners’ alleles^50^. For the mean number of amino acid substitutions, we assessed the average amino acid substitutions between the two partner alleles. For the maximum number, we selected the maximum value of amino acid substitutions. We also computed the pairwise genetic distance between the complete sequences of TLR genes for individuals of each breeding pair using MEGA11^82^. We calculated the total pairwise genetic distance for each of the four TLR loci and the total distance across all loci. We tested the association of our two genetic distance metrics (i.e. amino acid substitutions and pairwise genetic distance) with annual reproductive success (including year and pair identity as random variables) using GLMM (binomial error distribution and logit link), and with multi-year reproductive success using GLM (binomial error distribution and logit link). To facilitate model convergence, we standardized (centered and scaled) the pairwise genetic distance. Once again, we selected the best models by AICc for each set of models. We did not include pairwise genetic difference at *TLR1La* in our model list as we did not have enough data points.

## Electronic Supplementary Material

Below is the link to the electronic supplementary material.

Supplementary Material 1.

## Data Availability

The datasets generated during and analyzed during the current study are available on Figshare DOI: 10.6084/m9.figshare.24850827. Accession numbers from Genbank for the TLR loci are OR996376 to OR997657.
