# Supplementary material for: Differences between mates at the TLR1Lb locus are associated with lower reproductive success in a long-lived seabird
Source: Sci Rep. 2024 Dec 30;14:31608. doi: 10.1038/s41598-024-77750-7 (PMC11686313; doi:10.1038/s41598-024-77750-7)
Supplement: Supplementary file 1 — Supplementary Material 1. [file 41598_2024_77750_MOESM1_ESM.docx]

**SUPPLEMENTARY MATERIAL**

**Supplementary Table 1:** Model selection based on Akaike information Criterion (AICc) for the correlation between individual neutral genetic variation and **a)** annual orreproductive success and **b)** multi-year reproductive success. RS: reproductive success; RScomp: percentage of successful reproductive attempts; MLH: multi-locus heterozygosity; F: inbreeding coefficient. The most likely model is in bold.

| **a)** |  |  |  |  |
| --- | --- | --- | --- | --- |
| Models | AICc | Δ | AICc | AICcweight |
| **RS ~ 1 + (1\|Year) + (1\|BandNo)** | **475.18** | **0** | | **0.28** |
| RS ~ Age + (1\|Year) + (1\|BandNo) | 476.90 | 1.72 | | 0.12 |
| RS ~ F + (1\|Year) + (1\|BandNo) | 477.11 | 1.93 | | 0.11 |
| RS ~ MLH + (1\|Year) + (1\|BandNo) | 477.14 | 1.97 | | 0.11 |
| RS ~ F + Age + (1\|Year) + (1\|BandNo) | 477.18 | 2.01 | | 0.10 |
| RS ~ MLH^2^ + MLH + (1\|Year) + (1\|BandNo) | 478.85 | 3.67 | | 0.05 |
| RS ~ Sex + (1\|Year) + (1\|BandNo) | 478.90 | 3.72 | | 0.04 |
| RS ~ Sex + Age + (1\|Year) + (1\|BandNo) | 479.06 | 3.88 | | 0.04 |
| RS ~ MLH^2^ + MLH + Age + (1\|Year) + (1\|BandNo) | 479.13 | 3.95 | | 0.04 |
| RS ~ F + Sex + (1\|Year) + (1\|BandNo) | 479.16 | 3.98 | | 0.04 |
| RS ~ MLH + Sex + (1\|Year) + (1\|BandNo) | 480.67 | 5.49 | | 0.02 |
| RS ~ MLH + Sex + Age + (1\|Year) + (1\|BandNo) | 480.80 | 5.62 | | 0.02 |
| RS ~ MLH*Sex + (1\|Year) + (1\|BandNo) | 480.91 | 5.73 | | 0.02 |
| RS ~ MLH^2^ + MLH + Sex + (1\|Year) + (1\|BandNo) | 481.09 | 5.92 | | 0.01 |
| RS ~ MLH^2^ + MLH + Sex + Age + (1\|Year) + (1\|BandNo) | 482.83 | 7.66 | | 0.01 |
| **b)** |  |  |  |  |
| Models | AICc | Δ | AICc | AICcweight |
| **RScomp ~ 1** | **98.28** | **0** | | **0.38** |
| RScomp ~ Sex | 99.95 | 1.67 | | 0.17 |
| RScomp ~ F | 100.31 | 2.03 | | 0.14 |
| RScomp ~ MLH | 100.32 | 2.04 | | 0.14 |
| RScomp ~ F + Sex | 102.05 | 3.76 | | 0.06 |
| RScomp ~ MLH + Sex | 102.05 | 3.77 | | 0.04 |
| RScomp ~ MLH + MLH^2^ | 102.85 | 4.57 | | 0.04 |
| RScomp ~ MLH*Sex | 103.95 | 5.67 | | 0.02 |
| RScomp ~ MLH + MLH^2^ + Sex | 104.64 | 6.36 | | 0.02 |

**Supplementary Table 2**. Model selection based on Akaike information Criterion (AICc) for the correlation between individual functional genetic variation and **a)** annual reproductive success or **b)** multi-year reproductive success. RS: reproductive success; RScomp: percentage of successful reproductive attempts; MLH: multi-locus heterozygosity; PHt: proportion of heterozygous loci; HL: homozygosity by locus; IR: internal relatedness. The most likely model is in bold

| **a)** |  | | |  | |  |  | |
| --- | --- | --- | --- | --- | --- | --- | --- | --- |
| Models | AICc | | | Δ | | AICc | AICcweight | |
| RS ~ MLH + (1\|Year) + (1\|BandNo) | | 512.41 | 0.00 | | | | | 0.11 |
| RS ~ PHt + (1\|Year) + (1\|BandNo) | | 512.41 | 0.00 | | | | | 0.11 |
| RS ~ HL + (1\|Year) + (1\|BandNo) | | 512.41 | 0.00 | | | | | 0.11 |
| RS ~ IR + (1\|Year) + (1\|BandNo) | | 512.69 | 0.27 | | | | | 0.09 |
| **RS ~ 1 + (1\|Year) + (1\|BandNo)** | | **513.03** | **0.61** | | | | | **0.08** |
| RS ~ HL + Sex + (1\|Year) + (1\|BandNo) | | 513.40 | 0.98 | | | | | 0.07 |
| RS ~ MLH + Sex + (1\|Year) + (1\|BandNo) | | 513.40 | 0.99 | | | | | 0.07 |
| RS ~ PHt + Sex + (1\|Year) + (1\|BandNo) | | 513.40 | 0.99 | | | | | 0.07 |
| RS ~ IR + Sex + (1\|Year) + (1\|BandNo) | | 513.56 | 1.15 | | | | | 0.06 |
| RS ~ MLH + MLH^2^ + (1\|Year) + (1\|BandNo) | | 513.74 | 1.32 | | | | | 0.06 |
| RS ~IR*Sex + (1\|Year) + (1\|BandNo) | | 513.94 | 1.52 | | | | | 0.06 |
| RS ~ Sex + (1\|Year) + (1\|BandNo) | | 514.13 | 1.71 | | | | | 0.05 |
| RS ~ MLH + Sex + Age + (1\|Year) + (1\|BandNo) | | 514.30 | 1.89 | | | | | 0.04 |
| RS ~PHt*Sex + (1\|Year) + (1\|BandNo) | | 514.41 | 2.00 | | | | | 0.03 |
| RS ~MLH*Sex + (1\|Year) + (1\|BandNo) | | 514.41 | 2.00 | | | | | 0.03 |
| RS~HL*Sex + (1\|Year) + (1\|BandNo) | | 514.59 | 2.18 | | | | | 0.03 |
| RS ~ MLH + MLH^2^ + Sex + (1\|Year) + (1\|BandNo) | | 514.60 | 2.18 | | | | | 0.04 |
| RS ~ MLH + MLH^2^ + Age + (1\|Year) + (1\|BandNo) | | 514.66 | 2.24 | | | | | 0.04 |
| RS ~ MLH + MLH^2^ + Sex + Age + (1\|Year) + (1\|BandNo) | | 515.63 | 3.22 | | | | | 0.02 |
| **b)** | |  |  | |  | | |  |
| Models | | AICc | Δ | | AICc | | | AICcweight |
| **RScomp ~ 1** | | **162.91** | **0.00** | | | | | **0.48** |
| RScomp ~ Sex | | 164.88 | 1.97 | | | | | 0.18 |
| RScomp ~ MLH | | 164.92 | 2.01 | | | | | 0.17 |
| RScomp ~ MLH + MLH^2^ | | 166.49 | 3.58 | | | | | 0.08 |
| RScomp ~ MLH + Sex | | 166.92 | 4.02 | | | | | 0.06 |

**Supplementary Table 3.** Model selection based on Akaike information Criterion (AICc) for the correlation between heterozygosity at four TLR genes and **a)** annual reproductive success or **b)** multi-year reproductive success. RS: reproductive success; RScomp: percentage of successful reproductive attempts. The most likely model is in bold.

| **a)** |  |  |  |  |  |
| --- | --- | --- | --- | --- | --- |
| Models | AICc | Δ | AICc | AICcweight | |
| **RS~ 1 +(1\|Year) + (1\|BandNo)** | **513.03** | **0.00** | | **0.15** |  |
| RS~ TLR5 + (1\|Year) + (1\|BandNo) | 513.66 | 0.64 | | 0.11 |  |
| RS~ TLR1Lb + (1\|Year) + (1\|BandNo) | 514.05 | 1.02 | | 0.09 |  |
| RS ~ TLR1La + (1\|Year) + (1\|BandNo) | 514.17 | 1.15 | | 0.09 |  |
| RS ~ TLR1Lb + Sex + (1\|Year) + (1\|BandNo) | 514.90 | 1.87 | | 0.06 |  |
| RS ~ TLR4 + (1\|Year) + (1\|BandNo) | 514.93 | 1.90 | | 0.06 |  |
| RS ~ TLR5 + Sex + (1\|Year) + (1\|BandNo) | 514.94 | 1.91 | | 0.06 |  |
| RS ~ TLR1La + Sex + (1\|Year) + (1\|BandNo) | 515.22 | 2.20 | | 0.05 |  |
| RS ~ TLR1La*Sex + (1\|Year) + (1\|BandNo) | 515.80 | 2.77 | | 0.04 |  |
| RS ~ TLR5 + TLR1Lb + (1\|Year) + (1\|BandNo) | 516.01 | 2.99 | | 0.03 |  |
| RS ~ TLR4 +Sex + (1\|Year) + (1\|BandNo) | 516.02 | 2.99 | | 0.03 |  |
| RS ~ TLR1La + TLR1Lb + Sex + (1\|Year) + (1\|BandNo) | 516.02 | 2.99 | | 0.03 |  |
| RS ~ TLR5* Sex + (1\|Year) + (1\|BandNo) | 516.22 | 3.20 | | 0.03 |  |
| RS ~ TLR1Lb*Sex + (1\|Year) + (1\|BandNo) | 516.24 | 3.22 | | 0.03 |  |
| RS ~ TLR5 + TLR1La + (1\|Year) + (1\|BandNo) | 516.32 | 3.29 | | 0.02 |  |
| RS ~ TLR4 + TLR1Lb + Sex + (1\|Year) + (1\|BandNo) | 516.74 | 3.72 | | 0.02 |  |
| RS ~ TLR4 + TLR5 + (1\|Year) + (1\|BandNo) | 516.84 | 3.81 | | 0.02 |  |
| RS ~ TLR4*Sex + (1\|Year) + (1\|BandNo) | 517.15 | 4.12 | | 0.01 |  |
| RS ~ TLR1La + TLR1Lb + TLR4 + TLR5 + Sex + (1\|Year) + (1\|BandNo) | 517.82 | 4.79 | | 0.01 |  |
| **b)** |  |  |  |  |  |
| Models | AICc | Δ | AICc | AICcweight |  |
| **RScomp ~ 1** | **162.91** | **0.00** | | **0.30** |  |
| RScomp ~ TLR1La | 164.78 | 1.87 | | 0.12 |  |
| RScomp ~ TLR1Lb | 164.97 | 2.07 | | 0.11 |  |
| RSComp ~ TLR4 | 165.00 | 2.09 | | 0.10 |  |
| RScomp ~ TLR5 | 165.45 | 2.54 | | 0.08 |  |
| RScomp ~ TLR1La + Sex | 166.79 | 3.89 | | 0.04 |  |
| RScomp ~ TLR1Lb + Sex | 166.98 | 4.08 | | 0.04 |  |
| RSComp ~ TLR4 + Sex | 167.01 | 4.11 | | 0.04 |  |
| RScomp ~ TLR5 + Sex | 167.45 | 4.55 | | 0.03 |  |
| RScomp ~ TLR1La*Sex | 168.02 | 5.12 | | 0.02 |  |
| RScomp ~ TLR1La + TLR1Lb + Sex | 168.93 | 6.02 | | 0.01 |  |
| RScomp ~ TLR4 + TLR1La + Sex | 168.97 | 6.06 | | 0.01 |  |
| RScomp ~ TLR1Lb*Sex | 169.06 | 6.15 | | 0.01 |  |
| RScomp ~ TLR4 + TLR1Lb + Sex | 169.14 | 6.24 | | 0.01 |  |
| RSComp ~ TLR4*Sex | 169.15 | 6.24 | | 0.01 |  |
| RScomp ~ TLR1La + TLR5 + Sex | 169.38 | 6.48 | | 0.01 |  |
| RSComp ~ TLR5*Sex | 169.55 | 6.64 | | 0.01 |  |
| RScomp ~ TLR1Lb + TLR5 + Sex | 169.58 | 6.67 | | 0.01 |  |
| RScomp ~ TLR5 + TLR4 + Sex | 169.60 | 6.69 | | 0.01 |  |
| RScomp ~ TLR1La + TLR1Lb + TLR4 + TLR5 + Sex | 173.78 | 10.87 | | 0.00 |  |
|  |  |  |  |  |  |

**Supplementary Table 4.** Model selection based on Akaike information Criterion (AICc) for the correlation between number of amino acid substitutions at four TLR genes and total number of substitutions across the four loci and **a)** annual reproductive success or **b)** multi-year reproductive success. RS: reproductive success; RScomp: percentage of successful reproductive attempt; nb_sTot: total number of amino acid substitution across the four loci; nb_sTLR1La: a.a sub at TLR1La; nb_sTLR1Lb: a.a sub at TLR1Lb; nb_sTLR4: a.a sub at TLR4; nb_sTLR5: a.a sub at TLR5. The most likely model is in bold.

| **a)** |  |  |  |  |
| --- | --- | --- | --- | --- |
| Models | AICc | Δ | AICc | AICcweight |
| **RS ~ 1 + (1\|Year) + (1\|BandNo)** | **559.04** | **0.00** | | **0.15** |
| RS ~ nb_sTLR1La + (1\|Year) + (1\|BandNo) | 559.64 | 0.60 | | 0.11 |
| RS ~ nb_sTot + (1\|Year) + (1\|BandNo) | 560.30 | 1.25 | | 0.08 |
| RS ~ nb_sTLR1Lb + (1\|Year) + (1\|BandNo) | 560.52 | 1.48 | | 0.07 |
| RS ~ nb_sTLR4 + (1\|Year) + (1\|BandNo) | 560.55 | 1.51 | | 0.07 |
| RS ~ nb_sTRL1a + Sex + (1\|Year) + (1\|BandNo) | 560.66 | 1.62 | | 0.07 |
| RS~ nb_sTLR5 + (1\|Year) + (1\|BandNo) | 560.84 | 1.80 | | 0.06 |
| RS ~ nb_sTLR1Lb + nb_sTLR1La + (1\|Year) + (1\|BandNo) | 561.22 | 2.18 | | 0.05 |
| RS ~ nb_sTLR4 + nb_sTLR1La + (1\|Year) + (1\|BandNo) | 561.22 | 2.18 | | 0.05 |
| RS ~ nb_sTLR1Lb + Sex + (1\|Year) + (1\|BandNo) | 561.23 | 2.19 | | 0.05 |
| RS ~ nb_sTLR5 + nb_sTLR1La + (1\|Year) + (1\|BandNo) | 561.32 | 2.28 | | 0.05 |
| RS ~ nb_sTLR4 + Sex + (1\|Year) + (1\|BandNo) | 561.53 | 2.48 | | 0.04 |
| RS ~ nb_sTLR5 + Sex + (1\|Year) + (1\|BandNo) | 561.76 | 2.72 | | 0.04 |
| RS ~ nb_sTLR4 + nb_sTLR1Lb + (1\|Year) + (1\|BandNo) | 562.10 | 3.06 | | 0.03 |
| RS ~ nb_sTLR5 + nb_sTLR1Lb + (1\|Year) + (1\|BandNo) | 562.29 | 3.25 | | 0.03 |
| RS ~ nb_sTLR5 + nb_sTLR4 + (1\|Year) + (1\|BandNo) | 562.38 | 3.34 | | 0.03 |
| **b)** |  |  |  |  |
| Models | AICc | Δ | AICc | AICcweight |
| **RSComp ~ 1** | **162.91** | **0.00** | | **0.24** |
| RSComp ~ nb_sTot | 164.50 | 1.59 | | 0.11 |
| RSComp ~ nb_sTLR1La | 164.78 | 1.87 | | 0.09 |
| RSComp ~ nb_sTLR1Lb | 164.84 | 1.93 | | 0.09 |
| RSComp ~ nb_sTLR4 | 165.11 | 2.20 | | 0.08 |
| RSComp ~ nb_sTLR5 | 165.14 | 2.23 | | 0.08 |
| RSComp ~ nb_sTLR1Lb + nb_sTLR1La | 166.74 | 3.83 | | 0.04 |
| RSComp ~ nb_sTRL1a + Sex | 166.79 | 3.89 | | 0.03 |
| RSComp ~ nb_sTLR1Lb + Sex | 166.88 | 3.97 | | 0.03 |
| RSComp ~ nb_sTLR4 + nb_sTLR1La | 167.00 | 4.09 | | 0.03 |
| RSComp ~ nb_sTLR5 + nb_sTLR1La | 167.03 | 4.12 | | 0.03 |
| RSComp ~ nb_sTLR5 + nb_sTLR1Lb | 167.08 | 4.17 | | 0.03 |
| RSComp ~ nb_sTLR4 + nb_sTLR1Lb | 167.09 | 4.18 | | 0.03 |
| RSComp ~ nb_sTLR4 + Sex | 167.09 | 4.18 | | 0.03 |
| RSComp ~ nb_sTLR5 + Sex | 167.15 | 4.24 | | 0.03 |
| RSComp ~ nb_sTLR5 + nb_sTLR4 | 167.36 | 4.45 | | 0.03 |
|  |  |  |  |  |

**Supplementary Table 5.** Model selection based on Akaike information Criterion (AICc) for the correlation between neutral genetic variation differences between breeding partners on **a)** annual reproductive success or **b)** multi-year reproductive success. RS: reproductive success; RScomp: percentage of successful reproductive attempts; beta: beta relatedness estimate; Gen_sim; genetic relatedness. The most likely model is in bold.

| **a)** |  |  |  |  |
| --- | --- | --- | --- | --- |
| Models | AICc | Δ | AICc | AICcweight |
| RS ~ Gen_sim + (1\|Year) + (1\|PairID) | 120.37 | 0.55 | | 0.22 |
| **RS ~ 1 + (1\|Year) + (1\|PairID)** | **120.71** | **0.89** | | **0.19** |
| RS ~ Gen_sim + Gen_sim^2^ + (1\|Year) + (1\|PairID) | 122.17 | 2.35 | | 0.09 |
| RS ~ Gen_sim + Bird1_MLH + Bird2_MLH + (1\|Year) + (1\|PairID) | 123.76 | 3.94 | | 0.04 |
| RS ~ Gen_sim + Bird1_MLH* Bird2_MLH + (1\|Year) + (1\|PairID) | 123.77 | 3.96 | | 0.04 |
| RS ~ Bird1_MLH*Bird2_MLH + (1\|Year) + (1\|PairID) | 124.84 | 5.02 | | 0.02 |
| **b)** |  |  | |  |
| Models | AICc | Δ | AICc | AICcweight |
| **RScomp ~ 1** | **29.33** | **0.00** | | **0.45** |
| RScomp ~ Gen_sim | 31.16 | 1.83 | | 0.18 |
| RScomp ~ Gen_sim +Gen_sim^2^ | 33.20 | 3.87 | | 0.07 |
|  |  |  | |  |

**Supplementary Table 6.** Model selection based on Akaike information Criterion (AICc) for the correlation between functional genetic difference between breeding partners and **a)** annual reproductive success or **b)** multi-year reproductive success. RS: reproductive success; RScomp: percentage of successful reproductive attempts; Total_s: total number of amino substitutions between mates across the four loci; Mean_S: average amino substitution between mates for each locus; Max: maximum amino substitution between mates for each locus. The most likely model is in bold.

| **a)** |  |  |  |  | |
| --- | --- | --- | --- | --- | --- |
| Models | AICc | Δ | AICc | AICcweight |  |
| RS ~ Mean_STLR1La + (1\|Year) + (1\|Pair_ID) | 145.45 | 0.00 | | 0.14 |  |
| **RS ~ 1 + (1\|Year) + (1\|Pair_ID)** | **146.11** | **0.67** | | **0.10** |  |
| RS ~ MaxTLR1La + (1\|Year) + (1\|Pair_ID) | 146.28 | 0.83 | | 0.09 |  |
| RS ~ Total_s + (1\|Year) + (1\|Pair_ID) | 146.70 | 1.26 | | 0.07 |  |
| RS ~ Mean_STLR1La + Mean_STLR1Lb + (1\|Year) + (1\|Pair_ID) | 146.79 | 1.34 | | 0.07 |  |
| RS ~ Mean_STLR1La + Mean_STLR5 + (1\|Year) + (1\|Pair_ID) | 146.90 | 1.45 | | 0.07 |  |
| RS ~ Mean_STLR5 + (1\|Year) + (1\|Pair_ID) | 147.31 | 1.87 | | 0.06 |  |
| RS ~ Mean_STLR1La + Mean_STLR4 + (1\|Year) + (1\|Pair_ID) | 147.55 | 2.10 | | 0.05 |  |
| RS ~ Mean_STLR1Lb + (1\|Year) + (1\|Pair_ID) | 147.65 | 2.21 | | 0.05 |  |
| RS ~ MaxTLR4 + (1\|Year) + (1\|Pair_ID) | 148.11 | 2.66 | | 0.04 |  |
| RS ~ MaxTLR1Lb + (1\|Year) + (1\|Pair_ID) | 148.12 | 2.67 | | 0.04 |  |
| RS ~ Mean_STLR4 + (1\|Year) + (1\|Pair_ID) | 148.15 | 2.70 | | 0.04 |  |
| RS ~ MaxTLR5 + (1\|Year) + (1\|Pair_ID) | 148.19 | 2.75 | | 0.04 |  |
| RS ~ MaxTLR1La + MaxTLR1Lb + (1\|Year) + (1\|Pair_ID) | 148.26 | 2.82 | | 0.03 |  |
| RS ~ MaxTLR1La + MaxTLR4 +\|Year) + (1\|Pair_ID) | 148.33 | 2.88 | | 0.03 |  |
| RS ~ MaxTLR1La + MaxTLR5 + (1\|Year) + (1\|Pair_ID) | 148.38 | 2.93 | | 0.03 |  |
| RS ~ Mean_STLR1Lb + Mean_STLR5 + (1\|Year) + (1\|Pair_ID) | 148.77 | 3.33 | | 0.03 |  |
| RS ~ Mean_STLR4 + Mean_STLR5 + (1\|Year) + (1\|Pair_ID) | 149.38 | 3.93 | | 0.02 |  |
| RS ~ Mean_STLR4 + Mean_STLR1Lb + (1\|Year) + (1\|Pair_ID) | 149.74 | 4.29 | | 0.02 |  |
| RS ~ MaxTLR1Lb + MaxTLR4 + (1\|Year) + (1\|Pair_ID) | 150.17 | 4.73 | | 0.01 |  |
| RS ~ MaxTLR5 + MaxTLR4 + (1\|Year) + (1\|Pair_ID) | 150.21 | 4.76 | | 0.01 |  |
| RS ~ MaxTLR5 + MaxTLR1Lb + (1\|Year) + (1\|Pair_ID) | 150.22 | 4.77 | | 0.01 |  |
| RS ~ Mean_STLR4 + Mean_STLR5 + Mean_STLR1La + Mean_STLR1Lb + (1\|Year) + (1\|Pair_ID) | 150.23 | 4.79 | | 0.01 |  |
| RS ~ MaxTLR5 + MaxTLR4 + MaxTLR1La + MaxTLR1Lb + (1\|Year) + (1\|Pair_ID) | 152.50 | 7.05 | | 0.00 |  |
| **b)** |  |  |  |  | |
| Models | AICc | Δ | AICc | AICcweight | |
| **RSComp ~ MaxTLR1Lb** | **36.12** | **0.00** | | **0.19** |  |
| RSComp ~ MaxTLR1Lb + MaxTLR4 | 36.88 | 0.76 | | 0.13 |  |
| **RSComp ~ Mean_STLR1Lb** | **37.25** | **1.13** | | **0.11** |  |
| RSComp ~ Mean_STLR1La + Mean_STLR1Lb | 37.44 | 1.31 | | 0.10 |  |
| RSComp ~ MaxTLR5 + MaxTLR1Lb | 37.62 | 1.50 | | 0.09 |  |
| RSComp ~ Mean_STLR1Lb + Mean_STLR5 | 37.77 | 1.64 | | 0.08 |  |
| RSComp ~ Mean_STLR1Lb + Mean_STLR5 | 37.98 | 1.85 | | 0.07 |  |
| RSComp ~ MaxTLR5 + MaxTLR4 + MaxTLR1La + MaxTLR1Lb | 39.38 | 3.26 | | 0.04 |  |
| RSComp ~ Mean_STLR4 + Mean_STLR5 + Mean_STLR1La + Mean_STLR1Lb | 39.96 | 3.84 | | 0.03 |  |
| RSComp ~ Mean_STLR1La + Mean_STLR5 | 40.29 | 4.17 | | 0.02 |  |
| RSComp ~ MaxTLR5 | 41.18 | 4.37 | | 0.02 |  |
| RSComp ~ Mean_STLR5 | 41.30 | 5.06 | | 0.02 |  |
| RSComp ~ Mean_STLR1La | 41.53 | 5.17 | | 0.01 |  |
| RSComp ~ MaxTLR5 + MaxTLR4 | 41.62 | 5.40 | | 0.01 |  |
| RSComp ~ 1 | 41.87 | 5.50 | | 0.01 |  |
| RSComp ~ MaxTLR1La + MaxTLR5 | 41.88 | 5.75 | | 0.01 |  |
| RSComp ~ total_s | 42.29 | 5.76 | | 0.01 |  |
| RSComp ~ MaxTLR1La | 42.50 | 6.17 | | 0.01 |  |
| RSComp ~ MaxTLR4 | 42.51 | 6.37 | | 0.01 |  |
| RSComp ~ Mean_STLR4 + Mean_STLR5 | 42.97 | 6.39 | | 0.01 |  |
| RSComp ~ MaxTLR1La + MaxTLR4 | 43.31 | 6.85 | | 0.01 |  |
| RSComp ~ Mean_STLR4 + Mean_STLR1La | 43.33 | 7.19 | | 0.01 |  |
| RSComp ~ Mean_STLR4 | 43.40 | 7.28 | | 0.00 |  |

**Supplementary Table 7.** Model selection based on Akaike information Criterion (AICc) for the correlation between difference in functional variation between breeding partners and **a)** annual reproductive success or **b)** multi-year reproductive success. RS: reproductive success; RScomp: percentage of successful reproductive attempts; Total_s: total number of amino substitutions between mates across the four loci; Mean_S: average amino substitution between mates for each locus; Max: maximum amino substitution between mates for each locus. The most likely model is in bold.

| **a)** |  |  |  |  |
| --- | --- | --- | --- | --- |
| Models | AICc | Δ | AICc | AICcweight |
| **RS ~ Total_gen_dist + (1\|Year) + (1\|Pair_ID)** | **167.71** | **0.00** | | **0.31** |
| RS ~ Gen_dist_TLR1Lb + (1\|Year) + (1\|Pair_ID) | 169.71 | 1.99 | | 0.43 |
| RS ~ Gen_dist_TLR1Lb + Gen_dist_TLR5 + (1\|Year) + (1\|Pair_ID) | 169.84 | 2.12 | | 0.53 |
| RS ~ Gen_dist_TLR1Lb + Gen_dist_TLR4 + Gen_dist_TLR5 + (1\|Year) + (1\|Pair_ID) | 170.06 | 2.34 | | 0.63 |
| RS ~ Gen_dist_TLR1Lb + Gen_dist_TLR4 + (1\|Year) + (1\|Pair_ID) | 170.19 | 2.48 | | 0.72 |
| RS ~ 1 + (1\|Year) + (1\|Pair_ID) | 170.22 | 2.50 | | 0.81 |
| RS ~ Gen_dist_TLR5 + (1\|Year) + (1\|Pair_ID) | 170.53 | 2.82 | | 0.89 |
| RS ~ Gen_dist_TLR4 + (1\|Year) + (1\|Pair_ID) | 171.04 | 3.32 | | 0.95 |
| RS ~ Gen_dist_TLR5 + Gen_dist_TLR4 + (1\|Year) + (1\|Pair_ID) | 171.18 | 3.47 | | 1.00 |
| **b)** |  |  |  |  |
| Models | AICc | Δ | AICc | AICcweight |
| **RSComp ~ Gen_dist_TLR1Lb** | **36.41** | **0.00** | | **0.38** |
| RSComp ~ Gen_dist_TLR1Lb + Gen_dist_TLR5 | 37.3 | 0.9 | | 0.24 |
| RSComp ~ Gen_dist_TLR1Lb + Gen_dist_TLR4 | 38.43 | 2.02 | | 0.14 |
| RSComp ~ Gen_dist_TLR1Lb + Gen_dist_TLR4 + Gen_dist_TLR5 | 39.24 | 2.82 | | 0.09 |
| RS Comp~ Total_gen_dist | 40.29 | 3.88 | | 0.05 |
| RSComp ~ Gen_dist_TLR5 | 41.3 | 4.9 | | 0.03 |
| RSComp ~ 1 | 41.87 | 5.46 | | 0.02 |
| RSComp ~ Gen_dist_TLR5 + Gen_dist_TLR4 | 42.88 | 6.47 | | 0.02 |
| RSComp ~ Gen_dist_TLR4 | 43.32 | 6.92 | | 0.01 |

**Table 8.** PCR primers for TLR genes and sources.

|  |  |  |  |
| --- | --- | --- | --- |
| Gene | Primer | Sequence | Reference |
| *TLR1La* | avTLR1LaF | M13F-GATGGAATGAGCACTTCAGA | Alcaide & Edwards 2011 |
|  | avTLR1LaR | M13R-CTTCGTCTGCGTCCACTG | Alcaide & Edwards 2011 |
| TLR1Lb | PcaTLR1LBF | M13F-TCAGACTGTATGGCACTCATCC | Grueber et al 2013 |
|  | PcaTLR1LBR | M13R-TCAGCAGCAGAGCTGTCAC | Grueber et al 2013 |
| TLR2 | avTLR2F | M13F-AGGGACCTTCTGCACTCTG | Alcaide & Edwards 2011 |
|  | avTLR2R | M13R-AGGAGACAAAAGCGTCGTAG | Alcaide & Edwards 2011 |
| TLR4 | avTLR4F | M13F-GAGACCTTGATGCCCTGAG | Alcaide & Edwards 2011 |
|  | AplTLR4R | M13R-CCTGCCATCTTGAGCACTTG | Grueber et al 2013 |
|  |  |  |  |

**References**

Alcaide M, Edwards SV (2011) Molecular evolution of the toll-like receptor multigene family in birds. *Molecular Biology and Evolution*, **28**, 1703–15.

Grueber CE, Wallis GP, Jamieson IG (2013) Genetic drift outweighs natural selection at toll-like receptor (TLR) immunity loci in a re-introduced population of a threatened species. *Molecular Ecology*, **22**, 4470-4482.
